# Expression of FMRpolyG in Peripheral Blood Mononuclear Cells of Women with Fragile X Mental Retardation 1 Gene Premutation

**DOI:** 10.3390/genes13030451

**Published:** 2022-03-01

**Authors:** Xuan Phuoc Nguyen, Adriana Vilkaite, Birgitta Messmer, Jens E. Dietrich, Katrin Hinderhofer, Knut Schäkel, Thomas Strowitzki, Julia Rehnitz

**Affiliations:** 1 Department of Gynecological Endocrinology and Fertility Disorders, University Women’s Hospital, 69120 Heidelberg, Germany; xuanphuoc.nguyen@med.uni-heidelberg.de (X.P.N.); adriana.vilkaite@med.uni-heidelberg.de (A.V.); birgitta.messmer@med.uni-heidelberg.de (B.M.); jens.dietrich@med.uni-heidelberg.de (J.E.D.); thomas.strowitzki@med.uni-heidelberg.de (T.S.); 2 Institute of Human Genetics, Heidelberg University, 69120 Heidelberg, Germany; katrin.hinderhofer@med.uni-heidelberg.de; 3 Department of Dermatology, Heidelberg University, 69120 Heidelberg, Germany; knut.schaekel@med.uni-heidelberg.de

**Keywords:** *FMR1* premutation, FMRpolyG, premature ovarian insufficiency

## Abstract

Fragile X-associated primary ovarian insufficiency (FXPOI) is characterized by oligo/amenorrhea and hypergonadotropic hypogonadism and is caused by the expansion of the CGG repeat in the 5′UTR of Fragile X Mental Retardation 1 (*FMR1)*. Approximately 20% of women carrying an *FMR1* premutation (PM) allele (55–200 CGG repeat) develop FXPOI. Repeat Associated Non-AUG (RAN)-translation dependent on the variable CGG-repeat length is thought to cause FXPOI, due to the production of a polyglycine-containing *FMR1* protein, FMRpolyG. Peripheral blood monocyte cells (PBMCs) and granulosa cells (GCs) were collected to detect FMRpolyG and its cell type-specific expression in *FMR1* PM carriers by immunofluorescence staining (IF), Western blotting (WB), and flow cytometric analysis (FACS). For the first time, FMRpolyG aggregates were detected as ubiquitin-positive inclusions in PBMCs from PM carriers, whereas only a weak signal without inclusions was detected in the controls. The expression pattern of FMRpolyG in GCs was comparable to that in the lymphocytes. We detected FMRpolyG as a 15- to 25-kDa protein in the PBMCs from two *FMR1* PM carriers, with 124 and 81 CGG repeats. Flow cytometric analysis revealed that FMRpolyG was significantly higher in the T cells from PM carriers than in those from non-PM carriers. The detection of FMRpolyG aggregates in the peripheral blood and granulosa cells of PM carriers suggests that it may have a toxic potential and an immunological role in ovarian damage in the development of FXPOI.

## 1. Introduction

Premature ovarian insufficiency (POI) is a common endocrine reproductive disorder defined as a loss of ovarian activity before the age of 40 [1]. POI has a heterogeneous etiology, and a wide range of causes have been considered, including genetic, autoimmune, and iatrogenic [2]. Furthermore, 10–30% of idiopathic POI cases are genetic [3]. Fragile X-associated primary ovarian insufficiency (FXPOI), one of the most common genetic forms, is caused by the expansion of a CGG repeat in the 5′-untranslated region of the Fragile X mental retardation 1 (*FMR1*) gene [4].

FXPOI develops in approximately 20% of women carrying the premutation (PM) allele (55 to 200 unmethylated CGG repeats) [5], and 20% develop hypergonadotropic hypogonadism, compared with 1% of the general population [4]. Women with a PM with approximately 70–100 CGG repeats have the highest risk of FXPOI [6,7,8]. FXPOI is not observed in women with full mutations, occurring only in PM and, therefore, expanded CGG repeats in *FMR1* PM have been proposed as a pathogenic mechanism [4]. Several studies support this hypothesis by demonstrating increased *FMR1* mRNA levels in carriers of PM [9] and toxicity of the expanded CCG repeats [10,11,12].

Two main mechanisms have been proposed to explain the pathogenicity of expanded CGG repeats. In the first, the expanded CGG mRNA induces an RNA gain-of-function and sequesters specific RNA-binding proteins, preventing their cellular functions. A second mechanism is the repeat-associated non-AUG (RAN) translation via the expanded CGG repeat tract, which produces an FMR polyglycine-containing protein (FMRpolyG) [13,14].

RAN translation was first described by Zu et al. [15] as a process where expanded CAG repeats are translated in the absence of an AUG initiation codon in all three reading frames. RAN translation is initiated in the 5′-untranslated region (UTR) of the *FMR1* gene and produces FMRpolyG and FMR polyalanine (FMRpolyA) proteins [16]. RAN translation occurs in both PM (CGG: 59–160) and non-expansion (CGG: 30–50); however, aggregation is associated only with longer repeat tracts [17]. The presence of FMRpolyG in ubiquitin-positive inclusions has been demonstrated in *Drosophila* cells, mouse models, and the brain and other tissues from patients with FXTAS [16,18,19]. FMRpolyG protein expression is sufficient to cause cell death, decrease cell viability, disrupt the lamin ring, and form aggregates [15,16,19].

The colocalization of ubiquitin and FMRpolyG has recently been demonstrated in ovarian stromal and mural granulosa cells from *FMR1* PM carriers [18,20]. Shelly et al. [21] demonstrated impaired response to gonadotropin stimulation in two mouse models; one produced FMRpolyG and CGG-RNA, whereas the other only expressed CGG-RNA (99 CGG repeats). However, only the expression of both FMRpolyG and CGG-RNA resulted in a progressive loss of fertility with age. The exact molecular mechanisms of FXPOI development are still being explored, including the role of *FMR1* in ovarian function.

Recently, more studies have suggested that an association exists between *FMR1* PM and an increased risk of autoinflammatory and autoimmune disorders in female PM carriers [22,23]. These autoimmune disorders are thought to be related to RNA toxicity due to elevated *FMR1* mRNA levels in *FMR1* PM carriers [22,23,24]. Careaga et al. [25] reported deficits in immune responses in female PM carriers. They found that cytokine production and markers activated at the T cell surface (CD3+CD25+) were lower in *FMR1* PM carriers than in individuals with non-PM. Additionally, the CGG repeat length is associated with cytokine levels in both humans and mouse PM models.

Between 4 and 30% of all POI cases are of autoimmune origin [26,27]. There is evidence of an autoimmune etiology in POI, such as the detection of ovarian autoantibodies (AOAs), association with other autoimmune disorders, and the presence of lymphocytic oophoritis in biopsies [27]. Thus, the cellular immune function of PM carriers at a cellular level is still an important question.

Additionally, the lack of specific markers in female PM carriers who are still menstruating makes early diagnosis, risk assessment for FXPOI, and subsequent treatment challenging. Furthermore, the complex ovarian cell modeling process makes it difficult to study the role of these inclusions in FXPOI pathogenesis [20,28]. Therefore, this study aimed to detect FMRpolyG and its cell type-specific expression in peripheral blood monocyte cells (PBMCs) from *FMR1* PM carriers using immunofluorescence (IF) staining, Western blotting (WB), and flow cytometric analyses (FACS). Furthermore, we examined the potential usefulness of this protein as a biological marker for the early diagnosis of FXPOI in *FMR1* PM carriers.

## 2. Materials and Methods

### 2.1. Patients and Ethical Approval

Blood samples and granulosa cells from follicular punctures made during invitro fertilization (IVF)/intracytoplasmic sperm injection (ICSI) treatment were obtained from 10 control participants (aged 28–40 years) and five *FMR1* PM carriers who were still menstruating (aged 26–35 years) between October 2019 and May 2021 at Heidelberg University Women’s Hospital (Germany). All patients signed an informed consent form before participation and completed a clinical questionnaire.

This study was approved by the local ethics committee of the University of Heidelberg (number: S-602/2013) and was conducted according to the principles of the Declaration of Helsinki. Data on patient age, body mass index (BMI), and the expression of follicle stimulating hormone (FSH), luteinizing hormone (LH), estradiol (E2), and anti-Mullerian hormone (AMH) were collected and compared between both study groups. Hormone levels were determined as a basal profile on days 2–5 of a menstrual cycle. The determination was performed by our central clinical laboratory as follows: anti-Mullerian hormone (AMH), method—ECLIA, material—serum; follicle stimulating hormone (FSH), luteinizing hormone (LH), and estradiol (E2), method—immunoassay, material—heparin plasma.

### 2.2. CGG Repeat Length Analysis

Briefly, DNA samples were obtained from 10 mL blood samples collected in ethylenediaminetetraacetic acid (EDTA), as described previously [29]. The CGG repeat length of the 5′UTR of *FMR1* exon 1 was analyzed using polymerase chain reaction (PCR) analysis. Then, the region was sequenced using the ALFexpress DNA sequencer (Amersham 1050; Pharmacia Biotech, Freiburg, Germany) or the ABI 3100/3130xl sequencer (Life Technologies/Applied Biosystems, Foster City, CA, USA) as previously described [30]. When the presence of PM was suspected, a Southern blot analysis was performed using a 32P-dCTP radioactively labeled p2 probe containing *FMR1* exon 1 with CGG repeats, as described previously [31].

### 2.3. Gene Expression Analysis

Total RNA was extracted from leukocytes using the RNeasy mini kit (Qiagen, Hilden, Germany) as described previously [32]. For cDNA synthesis, total mRNA was reverse transcribed using oligo-(dT)15-primer and the M-MLV reverse transcriptase, RNase H Minus, Point Mutant (Promega, Madison, WI, USA) reverse transcription (RT). Gene expression assay kits for *FMR1* (Hs00924544_m1), two housekeeping genes predesigned by TaqMan (*HPRT* and *TBP* (Hs999909_m1 and Hs00427620_m1, respectively), and TaqMan Universal PCR Master Mix were purchased from Applied Biosystems (Life Technologies, Carlsbad, CA, USA). The experiments were performed according to the manufacturer’s instructions. Samples were analyzed in triplicate using standard PCR conditions with the 7500 Fast Real-Time PCR system (Applied Biosystems, Life Technologies, Carlsbad, CA, USA). *FMR1* mRNA expression was analyzed using the ΔΔCt method [15]. cDNA obtained from the COV 434 granulosa cells was used as a calibrator in each run.

### 2.4. PBMC Isolation

Whole blood was collected from women with and without *FMR1* PM into EDTA tubes. PBMCs were extracted using a density gradient with Ficoll-Paque^TM^ PLUS (Cytiva, Uppsala, Sweden) according to the manufacturer’s protocol. Blood samples were diluted (1:1) with Dulbecco’s phosphate-buffered saline (DPBS, Life Technologies, Grand Island, NY, USA) and carefully added to the same volume of Ficoll-Paque solution. Samples were centrifuged at 440× *g* for 30 min at room temperature (≅21 °C).

PBMCs at the interface were carefully isolated, washed twice with 1× PBS, and then IF-stained. For flow cytometric and WB analysis, cells were resuspended in cryopreservation medium (Roswell Park Memorial Institute (RPMI) 1640 medium (Life Technologies, Grand Island, NY, USA), 40% fetal bovine serum (FBS, Life Technologies, Grand Island, NY, USA), 10% dimethyl sulfoxide (DMSO, Serva, Heidelberg, Germany)) and stored at −80 °C.

### 2.5. IF Staining

PBMCs and human granulosa cells (hGCs) were IF-stained using established methods [33]. PBMCs were freshly collected and hGCs were collected from follicular fluid following oocyte retrieval, as previously described [34]. Cells were suspended in PBS, layered on glass coverslips at the bottom of the six-well culture plate and allowed to stand for 30 min to enable cells to adhere to the coverslip through gravity sedimentation. The adherent cells were fixed in 4% formaldehyde for 15 min and permeabilized with 0.2% PBS plus Triton X (PBST 0.2%) for 15 min.

The cells were blocked with 4% bovine serum albumin (BSA, Life Technologies, Grand Island, NY, USA) for 1 h to prevent nonspecific binding and then incubated overnight at 4 °C with the following primary antibodies: rabbit monoclonal antibody against ubiquitin (1:250, ab134953, Abcam, Cambridge, UK) and two novel mouse monoclonal antibodies against the N- and C-terminus of FMRpolyG 8FM (MABN2280) and 9FM (MABN1788; both 1:250, Merck, Darmstadt, Germany).

After washing with 1× PBS, the cells were incubated for 1 h at room temperature with the following secondary fluorescent antibodies: goat anti-rabbit Alexa Fluor Plus 555 (A21432) and goat anti-mouse Alexa Fluor Plus 488 (A32723; both 1:1000, Invitrogen, Carlsbad, CA, USA). The cells were washed three times, air dried, and mounted on slides using 4’,6-diamidino-2-phenylindole (DAPI)-containing mounting medium (P36931, Invitrogen, Carlsbad, CA, USA). Images were acquired using an EVOS M7000 microscope (Life Technologies, Bothell, WA, USA).

### 2.6. WB Analysis

PBMCs were lysed in M-PER™ (Pierce, Rockford, IL, USA) with 150 mmol/L sodium chloride (NaCl), 10 μg/mL chymostatin, 10 μg/mL antipain, and 1× Halt Protease and Phosphatase Inhibitor Cocktail (Pierce, Rockford, IL, USA). The cell debris was removed by centrifugation at 15,000× *g* for 15 min and the protein concentration was measured using a Bicinchoninic Acid (BCA) Protein Assay Kit (Pierce, Rockford, IL, USA). Proteins (50 ng) were electrophoretically separated using NuPAGE precast gels (4–12%, Bis-Tris, Invitrogen, Rockford, IL, USA) and transferred to 0.2 μm polyvinylidene fluoride (PDVF) membranes (Bio-Rad Laboratories Inc., Puchheim, Germany).

Membranes were incubated with anti-FMRpolyG 8FM (1:500), anti-FMRP (1:500, MA5-15499, Invitrogen, Carlsbad, CA, USA), anti-glyceraldehyde 3-phosphate dehydrogenase (GAPDH; 1:1000, PA1-987, Invitrogen, Carlsbad, CA, USA) overnight at 4 °C. Signals were visualized using SuperSignal West Femto Maximum Sensitivity Substrate (Thermo Fisher, Rockford, IL, USA) and imaged using the iBright CL 1500 Imaging System (Thermo Fisher, Singapore).

### 2.7. FACS Analysis

PBMCs were thawed on ice and washed with 1× PBS for flow cytometry. To exclude dead cells, the LIVE/DEAD Fixable Aqua Dead Cell Stain Kit (Life Technologies, Carlsbad, CA, USA) was used according to the manufacturer’s instructions. After pre-incubation with Fc Block (1:20; BioLegend, San Diego, CA, USA), cells were surface-stained with appropriate antibodies, CD3-PerCP, CD14-APC/Cy7 (1:100; BioLegend, San Diego, CA, USA), and CD19-APC (1:100; Miltenyi Biotec, Bergisch Gladbach, Germany) to identify T cells, B cells, and monocytes.

The cells were surface-stained, fixed with BD Cytofix/Cytoperm solution (BD Biosciences, San Diego, CA, USA) and then permeabilized with 1× BD Perm/Wash buffer (BD Biosciences, San Diego, CA, USA). The FMRpolyG 8FM antibody was conjugated using a fluorescein isothiocyanate (FITC) conjugation kit (Fast, ab188285, Abcam, Cambridge, UK) according to the manufacturer’s instructions and used at a concentration of 10 ng/mL for intracellular staining.

For ubiquitin antibodies that required labeling with a secondary antibody, cells were incubated with goat anti-rabbit Alexa Fluor Plus 555 (1:500, A21432, Invitrogen, Carlsbad, CA, USA) to complete immunostaining. The stained cells were washed and flow cytometry was performed using a Navios Flow Cytometer (Beckman Coulter, Krefeld, Germany). The mean fluorescence intensity (MFI) of FMRpolyG was determined using FlowJo software (version 10.7.2 for Mac OS X) for flow cytometric analysis. The gating strategy is shown in Supplemental Figure S3.

### 2.8. Statistical Analysis

The demographic data were compared using a two-tailed t-test (Fisher’s exact test) and all statistical analyses were performed using Prism 9.2.0 software. For all tests, a *p* < 0.05 was considered statistically significant.

## 3. Results

### 3.1. General Study Population

No differences were observed in our analyzed participants with and without PM in regards to age, BMI, and median hormone levels (FSH, LH, estrogen, and AMH). Consistent with previously reported studies of *FMR1* elevation in PM carriers, a significantly higher *FMR1* mRNA expression level was detected in the leukocytes of the PM group than in the control cells (*p* = 0.03; 5.2 ± 0.87 and 3.4 ± 0.36, respectively, Table 1).

### 3.2. FMRpolyG Aggregates Colocalized with Ubiquitin in PBMCs from FMR1 PM Carriers

**Figure 1:**
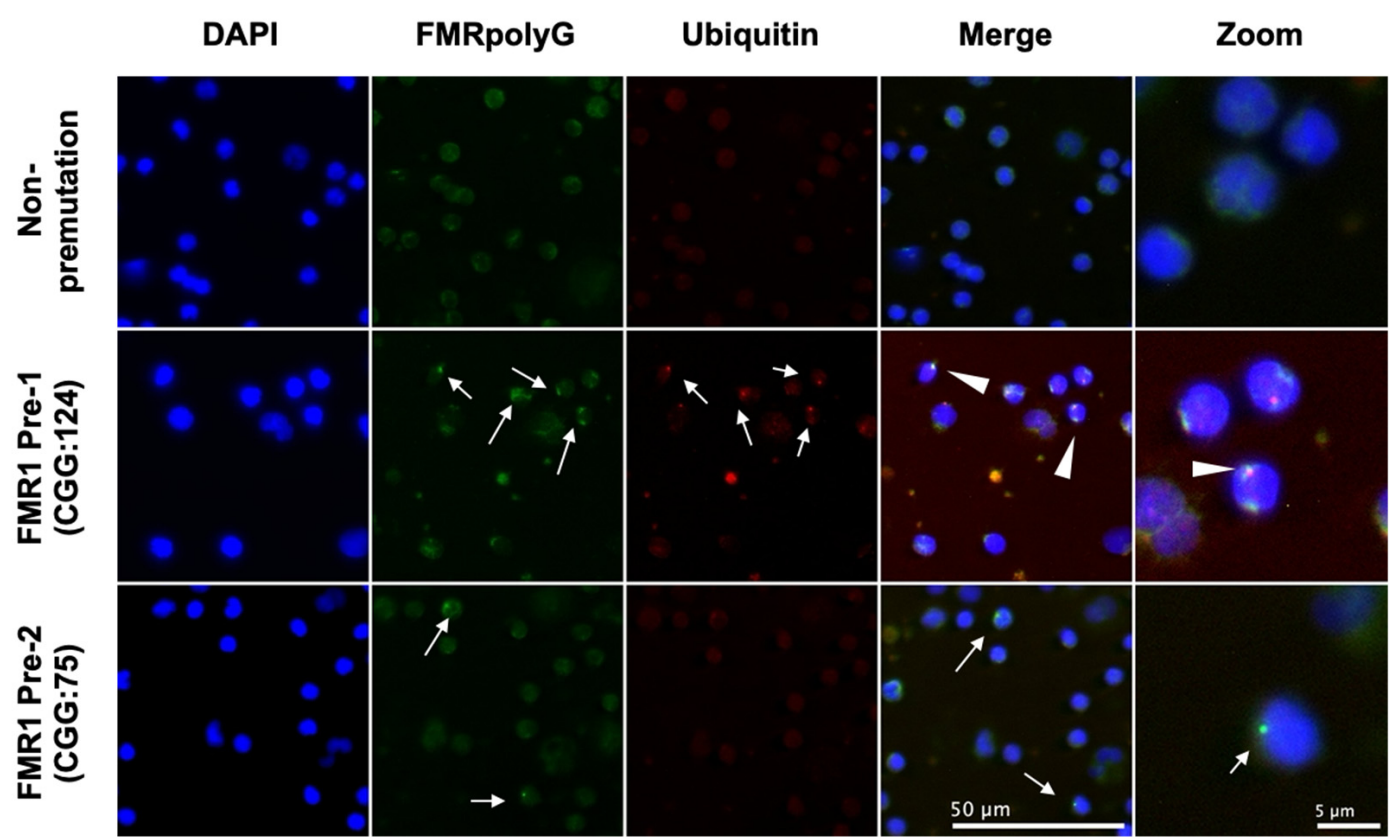
Fragile X mental retardation polyglycine (FMRpolyG) aggregates colocalized with ubiquitin in peripheral blood mononuclear cells (PBMCs) from *FMR1* gene premutation carriers. Immunofluorescence (IF) staining of FMRpolyG and ubiquitin in PBMCs. Cells were fixed and stained with FMRpolyG and ubiquitin antibodies. Cell nuclei were stained with 4’,6-diamidino-2-phenylindole (DAPI), (blue). Alexa Fluor 488 (green) and Alexa Fluor 555 (red) were used as secondary antibodies for FMRpolyG and ubiquitin, respectively. White arrows indicate the colocalization of FMRpolyG and ubiquitin. Scale bar, 50 μm.

IF staining of freshly isolated PBMCs detected FMRpolyG aggregates in samples from four of five *FMR1* PM carriers, whereas only a weak signal without inclusions was detected in all samples from women with normal CGG repeats. FMRpolyG aggregates colocalized with ubiquitin was detected in three patients, with higher CGG repeats (CGG: 124, 81, 78) than the others (Figure 1, Supplemental Figure S1). One patient with 75 CGG repeats showed no colocalization of FMRpolyG and ubiquitin in PBMCs (Figure 1). However, FMRpolyG aggregates were detected as ubiquitin-positive inclusions in the granulosa cells of this patient (Supplemental Figure S2). Furthermore, no FMRpolyG aggregates were detected in a patient with 72 CGG repeats.

### 3.3. FMRpolyG was Detected in PBMCs Lysate from FMR1 PM Carriers

**Figure 2:**
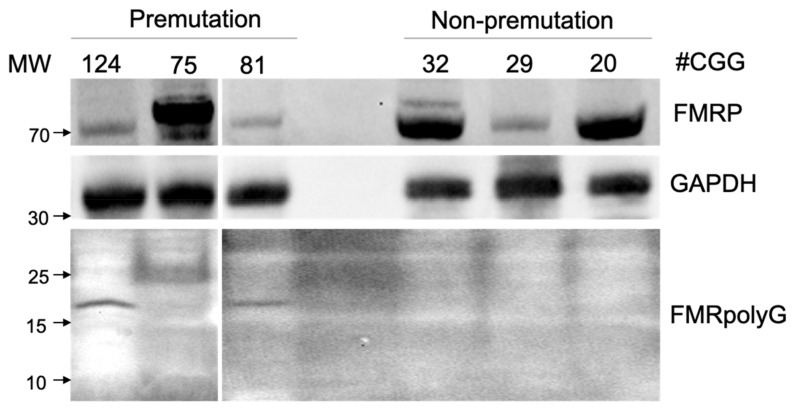
Fragile X mental retardation polyglycine (FMRpolyG) was detected in peripheral blood mononuclear cells (PBMCs) lysate from *FMR1* gene premutation carriers and age-matched individuals. FMRpolyG was detected in two *FMR1* premutation carriers (124 and 81 CGG repeats, respectively).

WB analysis to quantify the FMRpolyG expression in the PBMCs showed no expression in samples from women with non-PM. In contrast, FMRpolyG was found as a 15 to 25 kDa protein in two *FMR1* PM carriers, with 124 and 81 CGG repeats. The expression of FMRpolyG was more pronounced in patients with 124 CGG repeats than in those with 81 CGG repeats. FMRP expression was low in two FMRpolyG- positive patients (CGG: 124, 81; Figure 2).

### 3.4. Increase FMRpolyG Expression in T Cells from FMR1 PM Carriers

**Figure 3:**
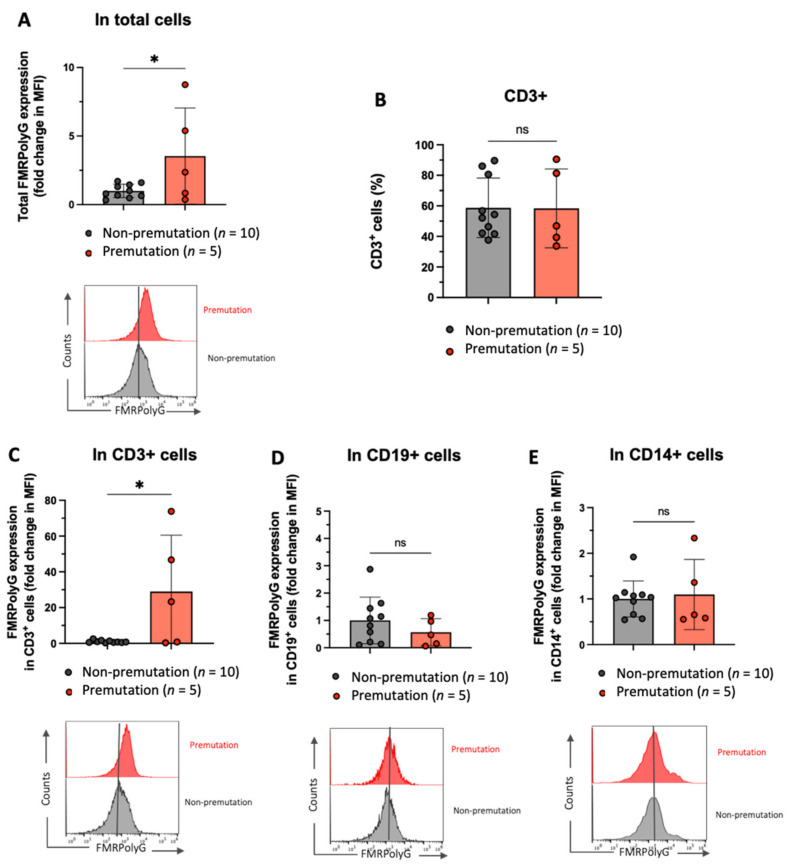
The increased fragile X mental retardation polyglycine (FMRpolyG) expression in T cells from *FMR1* gene premutation carriers. (**A**,**C**–**E**). The graph shows the fold change in mean fluorescent intensity (MFI), and the histograms show the FMRpolyG expression in total cells, T cells (CD3+), B cells (CD19+), and monocytes (CD14+). (**B**) The proportion of T cells in premutation (PM) and non-PM groups. *: *p* < 0.05 and ns: non-significant.

To comparatively analyze cell type-specific expression of FMRpolyG in PBMCs between women with *FMR1* PM and control participants without PM, we measured the FMRpolyG fluorescence intensity of three subsets of T cells (CD3+), B cells (CD19+), and monocytes (CD14+) via FACS. We found that FMRpolyG expression levels were significantly higher in the *FMR1* PM carriers than they were in the group without PM (3.5-fold, *p* = 0.03, Figure 3A). Both the *FMR1* PM and non-PM groups showed a normal distribution of T cells (58.38% ± 11.54 and 58.72% ± 6.16, respectively, Figure 3B).

However, the FMRpolyG fluorescence intensity was 28.98-fold higher (*p* = 0.01) in T cells (CD3+) from *FMR1* PM carriers than in those from women without PM (Figure 3C). FMRpolyG expression levels of B cells (CD19+) and monocytes (CD14+) were comparable in both groups (Figure 3D,E). The intracellular expression of total ubiquitin was significantly lower in cells from *FMR1* PM carriers than it was in those from the control group (Supplemental Figure S4A). Notably, ubiquitin-positive cells from *FMR1* PM carriers showed significantly higher expression levels of FMRpolyG than did those from non-PM carriers (17.84-fold, *p* < 0.0001, Supplemental Figure S4B).

## 4. Discussion

To the best of our knowledge, this is the first study to demonstrate the accumulation of FMRpolyG in the blood of *FMR1* PM carriers and its colocalization with ubiquitin. We also quantified the expression of solubilized FMRpolyG using WB analysis and found strong expression levels of FMRpolyG in T cells, suggesting a different role for FMRpolyG in FXPOI pathogenesis.

The discovery by Todd et al. [16] in 2013 that *FMR1* CGG repeats in mRNA cause RAN translation raises the possibility that FMRpolyG proteins contribute to the pathogenesis of FXTAS. Numerous studies have suggested the existence of pathophysiological similarities between FXPOI and FXTAS [5,35,36]. FMRpolyG protein has been detected in several animal models of FXTAS [19,37,38], along with FXTAS inclusions in patient samples [19,39] and in ovarian stromal cells from a woman with FXPOI [18]. In these samples, FMRpolyG was found in ubiquitin-positive intranuclear and perinuclear inclusions. Friedman-Gohas et al. [20] showed that FMRpolyG accumulates in granulosa cells from FMR1 PM carriers. Another RAN protein, poly-glutamine, has also been detected in the blood of patients with myotonic dystrophy type 1 (DM1) [40]. Furthermore, to the best of our knowledge, we are the first group to successfully detect FMRpolyG protein in the peripheral blood of women with *FMR1* PM.

We detected FMRPolyG-positive inclusions in four patients who had PM, but not in one patient with 72 CGG repeats. Numerous studies support the idea that CGG repeats can strongly influence transcriptional efficiency and thus, FMRpolyG production [9,41,42,43]. Sellier et al. [19] showed that expansion beyond 70 repeats is necessary for the detection of FMRpolyG aggregates, and FMRpolyG is a very stable protein with more than 90 glycines. These studies may explain why we observed a higher number of FMRpolyG aggregates in PBMCs from patients with higher expanded CGG repeats than in those with lower CGG repeats.

Recently, a large cross-sectional study of 1668 women by Allen et al. [44] showed that women with 85–89 CGG repeats were at the highest risk of developing FXPOI [44]. Our similar finding showing an increased expression of FMRpolyG aggregates in this CGG repeat range suggests that the expression of FMRpolyG inclusions in PBMCs could serve as a biomarker to facilitate the early diagnosis and subsequent treatment of FXPOI. In addition, FMRpolyG is reported to be translated from non-expanded lengths of CGG repeats (CGG: 30) [16,19] and to have a weaker expression in patient-derived control lymphoblasts (CGG:23) than in those with FXTAS (CGG: 100–117) [17]. Our result showed a similar fainter expression of FMRpolyG in the peripheral blood of women, without accumulation, in those who were non-PM than in those who were PM carriers.

We observed that colocalization of FMRpolyG with ubiquitin varied within the group of *FMR1* PM carriers. In FXTAS, FMRpolyG is primarily degraded via the ubiquitin-proteasome system (UPS), which may be dependent on FMRpolyG expression levels [15,45]. These studies may explain why we found more ubiquitin-positive inclusions in patients with 124 and 81 CGG repeats who had higher FMRpolyG, expression detected using WB, than in patients with lower expression levels. Moreover, we observed the same expression pattern of FMRpolyG aggregates and their colocalization with ubiquitin in the cytoplasm of *FMR1* PM granulosa cells as that reported by Friedman-Gohas et al. [20].

In contrast, we detected the weak expression of FMRpolyG in fresh granulosa cells from non-expanded CGG repeats, which was not found in the study by Friedman-Gohas et al. [20]. This may have been due to the rapid loss of the unstable FMRpolyG protein [19] during the first few days in the granulosa cell culture [46]. More studies with larger numbers of patients are needed to further elucidate the correlation between FMRpolyG expression in ubiquitin-positive inclusions and ovarian dysfunction. However, in this study, FMRpolyG accumulation appeared to be related to CGG repeat and the FMRpolyG expression level.

In this study, we confirmed the expression of FMRpolyG in PBMCs from *FMR1* PM carriers using WB analysis. Furthermore, our results were similar to those of previous studies that detected FMRpolyG in brain lysate samples from patients with FXTAS [19] and granulosa cells from patients who were PM carriers [20]. As expected, the *FMR1* mRNA transcript was significantly higher in the *FMR1* PM carriers than it was in the non-PM controls in our study. We also noted that the expression of FMRP was lower in the FMRpolyG-positive patients than it was in the group that did not express FMRpolyG and the control group.

These results are consistent with those of previous studies [9,16,30,43,47]. Our findings also suggest that CGG repeats play a role in regulating FMRP synthesis through the production of FMRpolyG and other proteins generated from an upstream open reading frame using a near-cognate AUG codon [16,17,48]. Therefore, the expression of FMRpolyG may contribute to the decreased FMRP level observed in *FMR1* PM carriers [19,20].

Our FACS data analysis was consistent with the immunofluorescence staining and immunoblotting results. The FMRpolyG fluorescence intensity is higher in the total PBMCs from females with *FMR1* PM and in cells with ubiquitin expression. Surprisingly, we observed a significant increase in FMRpolyG expression in T cells from PM carriers, even in the small number of patients in this study, whereas it did not increase in B cells or monocytes. Our results suggest a role for inflammatory and T cells in the pathological mechanism underlying FXPOI progression.

T cells play a central role in homeostasis and the host defense against infectious diseases [49,50]. In our study, T cells from *FMR1* PM carriers expressed a higher FMRpolyG fluorescence intensity than those from the controls, and the IF staining showed FMRpolyG accumulation in the immune cells. Previous studies have demonstrated the toxicity of FMRpolyG based on its reduction of cell viability, disruption of nuclear lamin architecture [15,16,19], and enhancement of stress protein expression [14].

Taken together, these findings led us to hypothesize that the increased expression of FMRpolyG in T cells may dysregulate the T cell homeostatic function in *FMR1* PM carriers. Dysregulated T cells are known to recognize self-antigens as foreign antigens, causing a harmful autoimmune response [49,51]. This altered T cell function may explain the decrease in cytokine production, the activated T cell surface markers [25], and the significantly increased rate of autoimmune and autoinflammatory disorders in carriers of the *FMR1* PM [52]. Interestingly, clinical autoimmune syndromes such as hypothyroidism, fibromyalgia, and irritable bowel syndrome mainly occur in female PM carriers [53], supporting our hypothesis.

The dysregulation of T cells may also cause inflammation in ovarian tissue [54,55,56]. Previous studies have suggested the involvement of neuroinflammation in the neurodegenerative processes in patients with FXTAS, which may enhance our understanding of the mechanisms mediating the progression of FXPOI. Ceredeno et al. discovered the inflammatory state in patients with FXTAS when they found signs of microglial cell activation and senescence in their brains [57]. Moreover, proinflammatory interleukin (IL)-12 and tumor necrosis factor (TNF)-α are elevated in the cerebellar tissue of those with FXTAS [58].

Peripherally, PBMCs isolated from men with PM have significantly higher levels of the anti-inflammatory cytokine (IL-10), and its concentration correlated with the number of CGG repeats [59]. Activated microglia have been reported to interact with T cells to promote neuroinflammation in other diseases [50,60]. Together, the findings of these studies provide evidence that sustained immune activation, possibly caused by T cells dysfunction, plays a causative role in FXTAS disease progression and may be found in the ovaries of patients with FXPOI. Further immunohistochemistry studies on oxidative stress and macrophage function in the ovarian tissue of FXPOI patients are needed.

## 5. Conclusions

For the first time, we found FMRpolyG accumulation in the peripheral blood and granulosa cells from *FMR1* PM carriers in the form of ubiquitin-positive inclusions. The expression of FMRpolyG was more pronounced in PM carriers with higher CGG repeats than it was in those with fewer repeats. These findings demonstrate the toxic potential of these protein fractions involved in ovarian damage in the development of FXPOI. The FMRpolyG expression level was significantly higher in the PM T cells than it was in the non-PM cells, which may indicate the immunological role of FMRpolyG in the pathological mechanism underlying FXPOI progression. Therefore, future experiments will be conducted on more patients to evaluate consistency and to elucidate the impact of FMRpolyG accumulation on fertility, along with the prospective value for individual ovarian preservation.

## Figures and Tables

**Table 1:** Clinical and laboratory characteristics of study participants: fragile X mental retardation 1 (FMR1) premutation (PM) and non (PM) groups.

	*FMR1* Non Premutation (*n* = 10)	*FMR1* Premutation (*n* = 5)	*p* Value
Mean Age (SD)	34.20 (1.29)	31.00 (1.51)	0.15
Mean BMI (SD)	24.31 (0.75)	23.06 (1.30)	0.38
Median FSH (U/L) (SD)	10.04 (6.0–98.4)	12.01 (9.2–29.5)	0.61
Median LH (U/L) (SD)	4.75 (2.9–39.2)	6.3 (2.2–28.6)	0.61
Median Estradiol (pg/mL) (SD)	37.55 (23.8–75.5)	52.8 (33.8–111.4)	0.12
Median AMH (ng/mL)	1.48 (0.83–2.87)	0.89 (0.79–2.8)	0.56
Mean *FMR1* repeats (range)	27.60 (20–32)	86 (72–124)	
*FMR1* mRNA expression level in lymphocytes	3.4 (0.36)	5.2 (0.87)	0.03

## Data Availability

Data are available upon reasonable request from the corresponding author.

## Supplementary Materials

The following supporting information can be downloaded at https://www.mdpi.com/article/10.3390/genes13030451/s1: Figure S1—Fragile X mental retardation polyglycine (FMRpolyG) and ubiquitin expression in peripheral blood mononuclear cells (PBMCs) from *FMR1* premutation (PM) carriers and non-PM participants; Figure S2—Fragile X mental retardation polyglycine (FMRpolyG) aggregates colocalized with ubiquitin in human granulosa cells (hGCs) from *FMR1* gene premutation carrier (75 CGG repeats); Figure S3—Flow cytometry gating strategy to identify three monocyte subsets and ubiquitin from peripheral blood mononuclear cells (PBMCs); Figure S4—Graph showing fold change in mean fluorescent intensity (MFI) and histograms showing ubiquitin expression in total cells (A) and FMRpolyG expression in ubiquitin positive cell (B).
