# Supplementary material for: Expression of FMRpolyG in Peripheral Blood Mononuclear Cells of Women with Fragile X Mental Retardation 1 Gene Premutation
Source: Genes (Basel). 2022 Mar 1;13(3):451. doi: 10.3390/genes13030451 (PMC8951797; doi:10.3390/genes13030451)
Supplement: Supplementary file 1 [file genes-13-00451-s001.zip › genes-1613542-supplementary.pdf]

genes
MDPI

Supplementary Materials

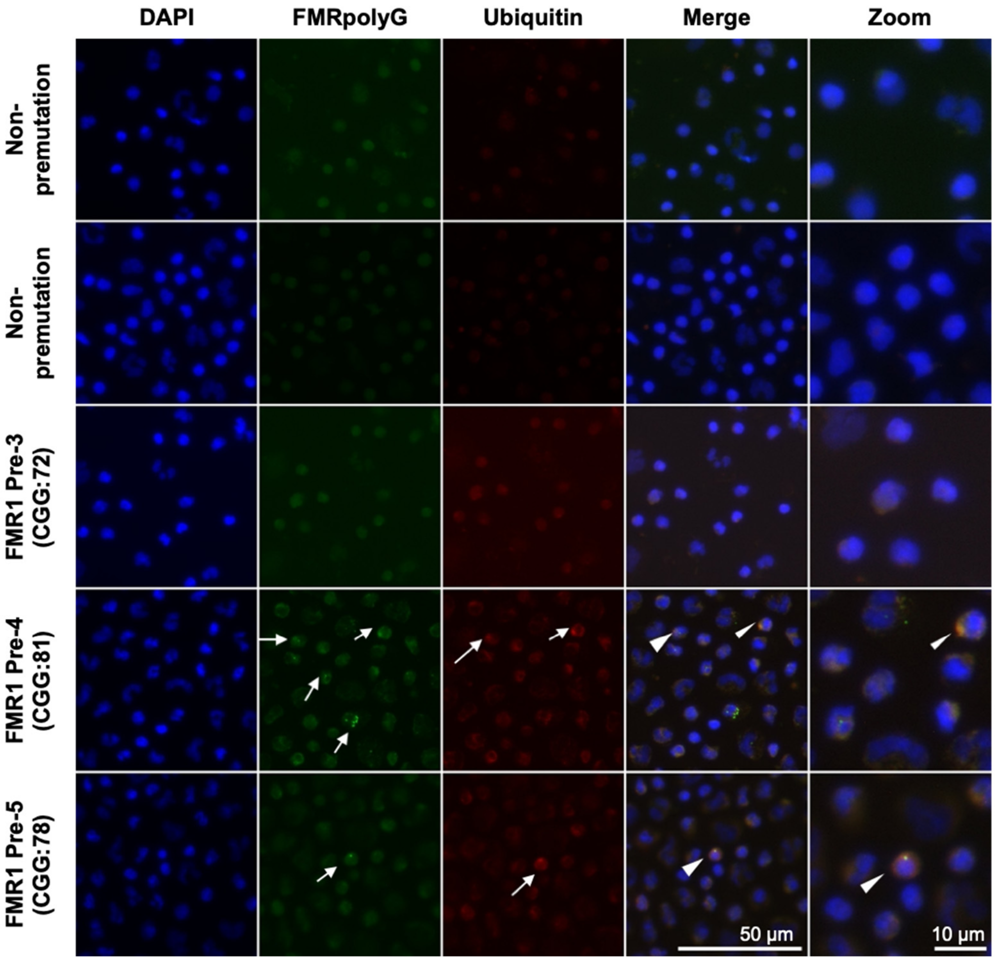

Figure S1. FMRpolyG and ubiquitin expression in PBMCs from *FMR1* premutation carriers and non premutation. Immunofluorescence staining of FMRpolyG, ubiquitin in PBMCs. Cells were fixed and stained with FMRpolyG and ubiquitin. Cell nuclei were stained with DAPI (blue). Alexa 488 (green) and Alexa 555 (red) were used as secondary antibodies for FMRpolyG and ubiquitin, respectively. The white arrowheads indicate the co-localization of FMRpolyG and ubiquitin. Scale bar, 50 µm.

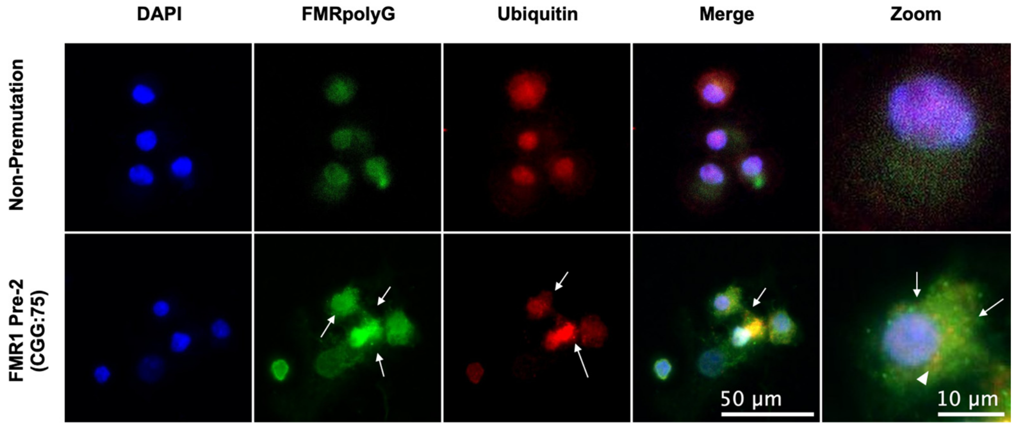

Figure S2. FMRpolyG aggregates colocalized with ubiquitin in human granulosa cells from *FMR1* premutation carrier (75 CGG repeats). Immunofluorescence staining of FMRpolyG, ubiquitin in human granulosa cells. Cells were fixed and stained with FMRpolyG and ubiquitin. Cell nuclei were stained with DAPI (blue). Alexa 488 (green) and Alexa 555 (red) were used as secondary antibodies for FMRpolyG and ubiquitin, respectively. The white arrows indicate the co-localization of FMRpolyG and ubiquitin. Scale bar, 20μm.

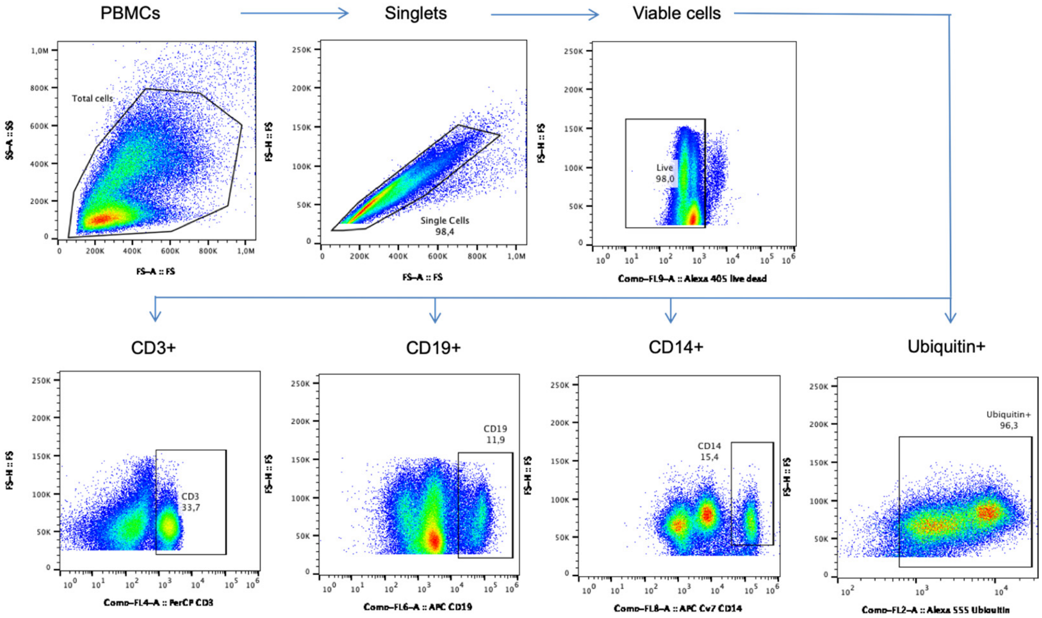

Figure S3. Flow cytometry gating strategy to identify the three monocyte subsets and ubiquitin from PBMCs.

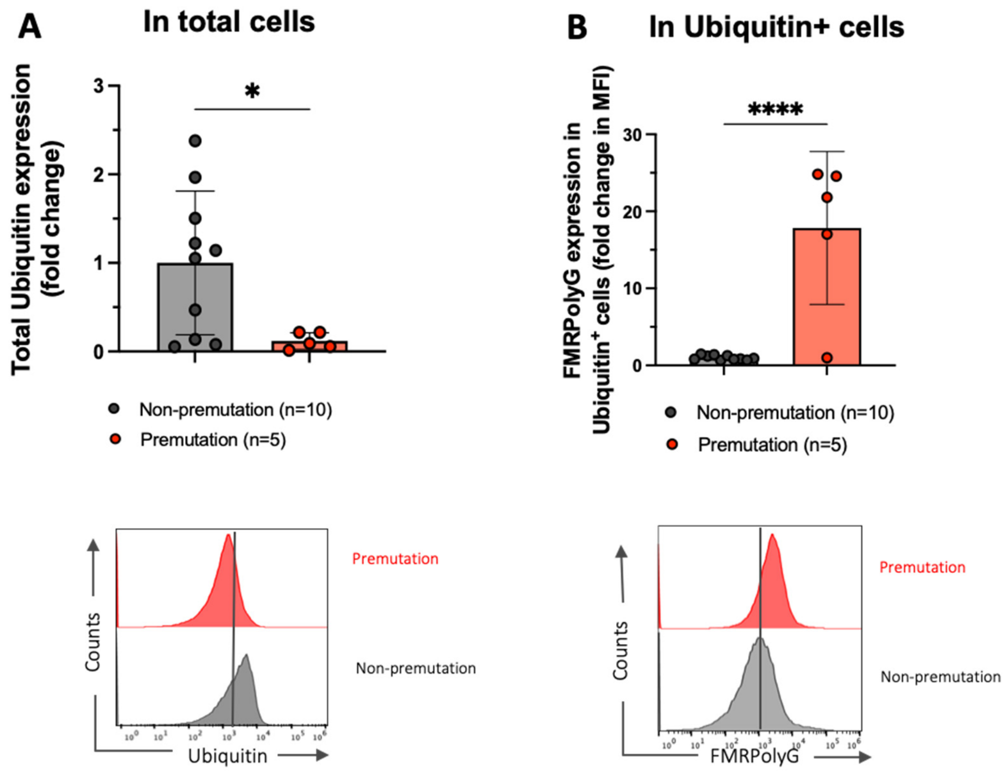

Figure S4. Graph showing fold change in MFI and histograms showing ubiquitin expression in total cells (**A**) and FMRpolyG expression in ubiquitin-positive cell (**B**).
